# The Present Status of Surgical Cleanliness

**Published:** 1917-09

**Authors:** Southgate Leigh

**Affiliations:** Norfolk, Va.; Surgeon-in-Charge, Sarah Leigh Hospital


### THE PRESENT STATUS OF SURGICAL CLEANLINESS.

**By Southgate Leigh, M.D., F.A.C.S., Norfolk, Va.**
Surgeon-in-Charge, Sarah Leigh Hospital.

I am sure this audience has been more or less surprised at my selecting for the subject of this talk a matter considered nowadays so elementary that but little attention is paid to it in the text-books or journals. In the light of present-day knowledge which the profession has to be guided by, the principles and rules of surgical cleanliness are indeed so simple that a child can easily understand and carry them out.

Then why do we feel justified in bringing it to your attention to-night? For the simple reason that to the impartial observer it would seem that notwithstanding its vital importance and that it is practically the foundation stone of modern technic, the carrying out of surgical cleanliness in operative work has taken a step backward during the past two or three years.

What are our reasons for making so serious a statement?

In a conversation recently with a prominent leader and teacher of surgery in a large city not far north of us, we were astounded to hear him say that he recently was unfortunate enough to lose a clean case of breast amputation from streptococcic infection. This occurred in a large, well-equipped hospital and the cause of infection had not been discovered.

At the Lynchburg meeting of the State Society one of the best surgeons of the country, and a pioneer in aseptic and antiseptic surgery, related a fatal case of streptococcic infection following a clean operation on the bones of the forearm. He attributed the trouble to improperly sterilized gloves.

Most of us here present have from time to time visited the various large surgical clinics, where we have ofttimes seen the most perfect and original operative work. How often is this brilliant work marred by infection!

Look through the various journals of current medical literature, and note how frequently infection is mentioned as an unfortunate complication.

Now, is this right? If surgical cleanliness is so simple and so easy to carry out, why is it not properly practiced?

We all know that the surgeon to-day is invading parts of the body which the old time surgeon did not dare to touch, and he is doing so because surgical cleanliness makes it safe.

We also know that any carelessness in the carrying out of clean technic is liable at any time and with any case to produce the horrible results that formerly occurred before surgeons knew how to be clean.

We have always felt very strongly on this subject. We believe that a surgeon is responsible for the operation and everything and everybody connected with it.

Infecting clean wounds, is an awful occurrence; the surgeon is the responsible one and he cannot transfer the responsibility to any one else.

The Golden Rule is a mighty good rule for the surgeon to keep constantly in mind and to constantly follow. Have we a right to do any work for a patient that we would not be willing to have done on ourselves? If we always put ourselves in the patient's place before determining on our course of action, wouldn't there be fewer operations done? Wouldn't we investigate our cases more vigorously and in a more deliberate manner? Wouldn't there be fewer exploiting laparotomies and fewer gastro-enterostomies, for example? And in the same manner, if any one of us here had to be operated on, he would want to be as certain as is possible with human knowledge that there would be no chance of infecting his wound and system with dangerous organisms.

If surgical cleanliness is so simple, why is it that we are constantly hearing of clean wounds being infected?

The causes of this unfortunate state of affairs, which the profession seems to carelessly view as a matter of course, are manifold. Let us consider a few of them.

The average operator does not realize the danger of carelessness, nor the necessity of extreme watchfulness. He takes what is put before him and does the best he can himself, feeling that if anything goes wrong he can't help it. He does not watch his assistants and nurses closely, nor look after the large number of preparatory details that go to make a clean operation. He takes it for granted that anybody and everything is clean and does not find it out positively.

The mixing of dirty and clean cases is often risky. The plan formerly followed by some careful men of having separate operating rooms for clean and dirty cases would probably be best for the average hospital.

The careless and thoughtless way of throwing and spreading dirty discharges and dirty sponges about the operating room is a source of danger.

The most serious problem in surgical cleanliness to-day is the question of gloves. Probably most of the infection in the large hospitals can be traced to gloves. This subject we will take up again later.

The hands of surgeons and assistants are often neglected by thoughtlessly handling dirty cases, ungloved, and making dirty examinations.

Many men are going into surgery in recent years without proper training. There is a good deal of vitally important knowledge that cannot be learned from text-books or journals or even from the clinics. Many of the details of successful surgery can only be gotten in a training school for surgeons, a good hospital, under a good surgical guide. The man who goes into surgery without much training, does not realize what he has missed.

Cleanliness in surgery is one of those important matters of detail which can be learned thoroughly in no other way. It should be as natural to the surgeon as breathing, and any breach of its principles should appear at once as a violent shock.

One doing surgery, who has not served the essential apprenticeship, does not notice the errors that may be made by others, and frequently when off guard he may make "slips" himself.

If our ideas are correct, that the profession is possibly going backward in this most important and vital matter, what remedy have we to offer? For surely this state of affairs should not be allowed to continue. Every conscientious man should make up his mind not to rest until clean surgery can be done without infections.

In considering this part of our subject we shall take first the work of the surgeon and next the work of the general practitioner.

The surgeon must have absolute control of his staff, including the operating room nurses. He must instruct them minutely in all of the necessary details, and impress upon them vigorously the extreme danger of any relaxation in strictness. This instruction should be repeated from time to time. He should not stop there, but, in addition, should watch each and every one on every possibly occasion. It is a mighty good plan to say to a nurse, "You may be the next one to be operated on; would you like to be infected?"

The ideal plan, of course, would be for each surgeon to have his own operating room and operating staff, even in the general hospitals. In this way the training and examples of the careful surgeon will not be counteracted by the bad example of the bad one. Is it not strange how assistants are so apt to follow the bad, rather than the good example?

Eventually surgeons are coming to this plan. It will really be forced upon them, if they wish to be positive and exact about their work.

As stated before, the glove problem is the serious and more or less uncertain one. Punctures must be looked after with extreme strictness. Gloves will not stand sufficient boiling and the boiling is rarely done properly, because of the air being left in the gloves. A glove used in a dirty case should never, under any circumstances, be used afterward in a clean case. To positively avoid such an error, we glue a small red rubber patch on the wrist of all such gloves.

After as careful and thorough boiling as possible the glove is put on, and the gloved hand is then soaked by us in 1 to 250 bichlorid, and this washed off with a weaker solution.

If the operator's hands are rough we advocate the wearing of two pairs of gloves.

No surgeon, assistant or surgical nurse should ever handle dirty wounds or discharges or make dirty examinations, such as mouth, vaginal or rectal, with the ungloved hand.

The operating room should be free of dust and as free of furniture as possible. No one should be allowed to enter the operating room ungowned.

Every drop of pus should be caught and immediately destroyed. If unavoidably the table or floor should be soiled, the soiled spots must be cleansed at once with a strong antiseptic. The throwing of sponges, pads, etc., about the floor is out of the question.

Without this strict care of septic discharge it cannot be safe to do clean surgery in the same room. Very badly infected cases should be treated elsewhere. If unavoidably they are operated on in the general operating room, fumigation should follow the operation.

No one in the operating department should be allowed to handle pus or anything soiled with septic matter, except with instruments or with gloved hands, and preferably the former.

The same principles regarding pus should be followed in the rooms and wards, and if a discharge shows through a dressing it should be immediately covered. This catching and destroying of septic materials can be easily done and will result in a very much cleaner hospital.

The general practitioner has to deal with surgical cleanliness in his minor surgery and obstetrics.

We would urge the more general use of gloves in examinations, dressings and obstetrics.

In our own recollection, and in this city, prominent and successful men have had to give up all obstetrical engagements on account of epidemics of puerperal fever. That was before the days of gloves. The general practitioner should keep his hands clean by using gloves in examinations and all dirty dressings, and he would also find the instrumental dressings of wounds to be of material aid.

While sterilized gloves ought to be used always in obstetrics, and we would urge such use, yet as a matter of fact they are not popular. There is one thing, however, that we would insist upon as being vitally necessary, and that is that the doctor who will not apply gloves in this way, should use them in all septic cases, especially puerperal.

Obstetrical cases should be treated as surgical and the strictness in cleanliness should be extreme. Much carelessness is still noted in this work The excuse is lack of time and poor assistance. There is no excuse, however, for not bathing the case thoroughly with soap and water and an antiseptic solution. If sterile sheets and towels are not available, it is always easy to surround the parts with wet bichlorid towels, especially if the side position be used for the delivery.

Men doing obstetrics ought to be stricter in cleanliness. It is at times noted that in the use of forceps the operator, after properly cleaning his hands and patient, will thoughtlessly move a chair or place the forceps on something unsterile.

No thought of criticism of any of our hearers has been intended in these remarks.

Our ideal of the local medical meetings is that we should each one do his part in the work of mutual benefit and improvement.

This paper, imperfect and hurriedly written, has been presented with the earnest hope that it may do some little towards increasing our interest in the vital and ever important subject of surgical cleanliness.—The International Journal of Surgery.

